# Supplementary figures and images for: Enhancing detection of labor violations in the agricultural sector: A multilevel generalized linear regression model of H-2A violation counts
Source: PLoS One. 2024 May 17;19(5):e0302960. doi: 10.1371/journal.pone.0302960 (PMC11101028; doi:10.1371/journal.pone.0302960)

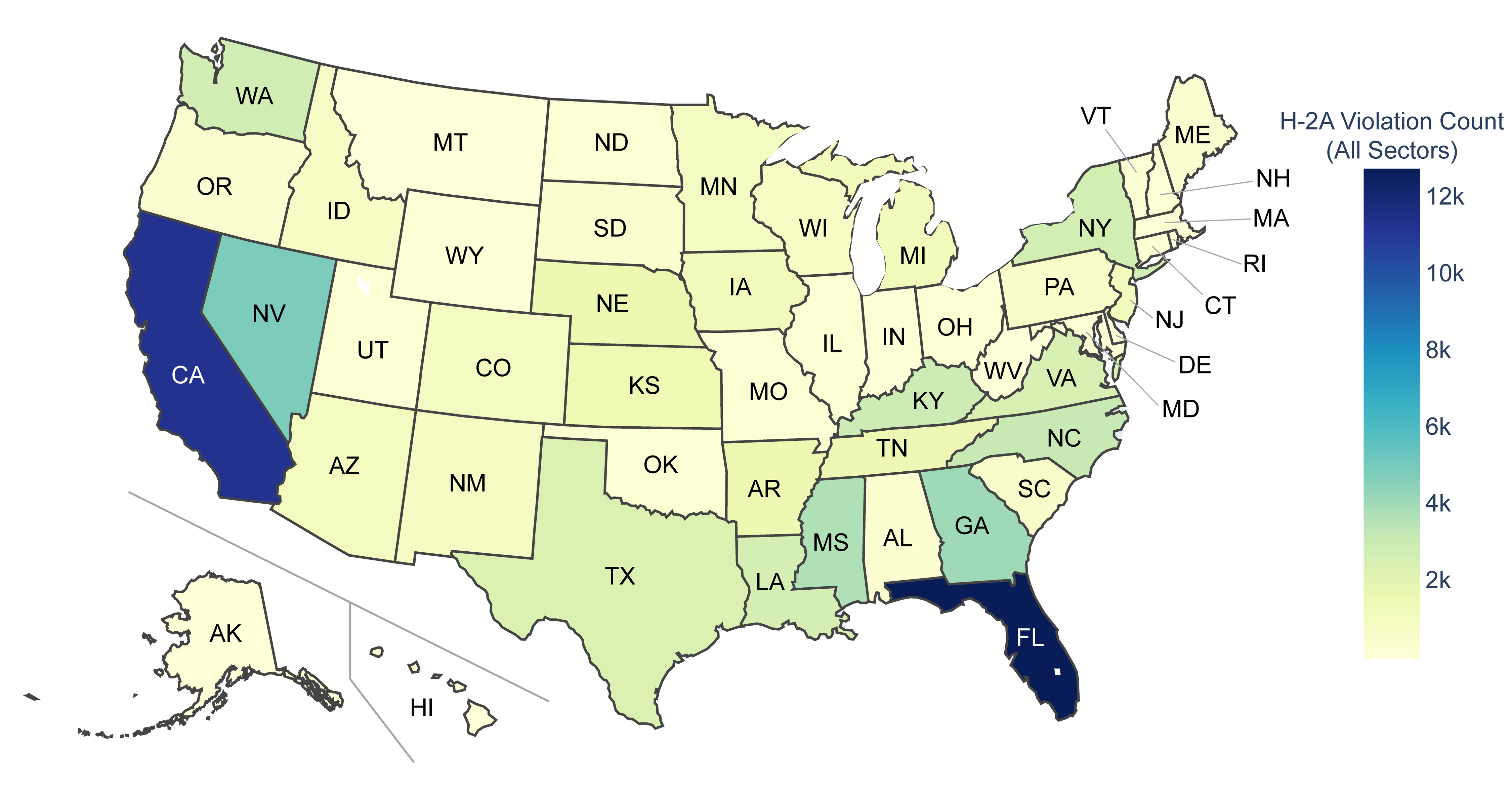

Supplement: S1 Fig — The heatmap illustrates the total number of identified H-2A violations per state from 2010—2020. Note that these violations span various industries, not just those with NAICS codes beginning with “11”. (TIF) [file pone.0302960.s001.tif]
