# Supplementary material for: Enhancing detection of labor violations in the agricultural sector: A multilevel generalized linear regression model of H-2A violation counts
Source: PLoS One. 2024 May 17;19(5):e0302960. doi: 10.1371/journal.pone.0302960 (PMC11101028; doi:10.1371/journal.pone.0302960)
Supplement: S1 File — (DOCX) [file pone.0302960.s002.docx]

**S1 Data Availability:**

The data used in this study can be found via this link:

<https://github.com/Arezoojafari/Enhancing-Detection-of-Labor-Violations>
